# Effect of the KF post-deposition treatment on grain boundary properties in Cu(In, Ga)Se_2_ thin films

**DOI:** 10.1038/srep41361

**Published:** 2017-01-27

**Authors:** N. Nicoara, Th. Lepetit, L. Arzel, S. Harel, N. Barreau, S. Sadewasser

**Affiliations:** 1 INL – International Iberian Nanotechnology Laboratory, Av. Mestre José Veiga s/n, 4715-330 Braga, Portugal; 2 Institut des Matériaux Jean Rouxel (IMN) - UMR6502, Université de Nantes, CNRS, 2 rue de la Houssinière, BP 32229, 44322 Nantes Cedex 3, France

## Abstract

Significant power conversion efficiency improvements have recently been achieved for thin-film solar cells based on a variety of polycrystalline absorbers, including perovskites, CdTe, and Cu(In,Ga)Se_2_ (CIGS). The passivation of grain boundaries (GBs) through (post-deposition) treatments is a crucial step for this success. For the case of CIGS, the introduction of a potassium fluoride post-deposition treatment (KF-PDT) has boosted their power conversion efficiency to the best performance of all polycrystalline solar cells. Direct and indirect effects of potassium at the interface and interface-near region in the CIGS layer are thought to be responsible for this improvement. Here, we show that also the electronic properties of the GBs are beneficially modified by the KF-PDT. We used Kelvin probe force microscopy to study the effect of the KF-PDT on the CIGS surface by spatially resolved imaging of the surface potential. We find a clear difference for the GB electronic properties: the KF-PDT increases the band bending at GBs by about 70% and results in a narrower distribution of work function values at the GBs. This effect of the KF-PDT on the GB electronic properties is expected to contribute to the improved efficiency values observed for CIGS thin-film solar cells with KF-PDT.

Harvesting of abundant sun light through photovoltaic (PV) power conversion is considered to be a cornerstone of the renewable energy supply for a sustainable future. Presently, Si wafer-based solar modules dominate the market. Nevertheless, polycrystalline thin-film PV technologies present substantial advantages, including potential for lower costs, for flexible substrates, and for reduced material consumption and weight. Despite the abundance of grain boundaries (GBs) in thin-film solar cells based on Cu(In,Ga)Se_2_ (CIGS), CdTe, and the recent perovskite absorbers, all of these materials have achieved power conversion efficiencies above 22%^1^,^2^, within reach of that of single crystalline silicon. The physics and the role of GBs in these materials has therefore received considerable attention. In CdTe solar cells, it has been shown that the CdCl_2_ post-deposition treatment leads to the passivation of GBs^3^,^4^. For organometal trihalide perovskite solar cells, treatment with a PCBM/C_60_ double fullerene layer was shown to passivate defects at GBs, thereby eliminating photocurrent hysteresis effects^5^. GB passivation has been shown to reduce non-radiative recombination and improve device efficiencies^6^,^7^.

For polycrystalline CIGS solar cells the role and physics of GBs has been intensively investigated. Cu depletion at GBs has been proposed to cause a lowering of the valence band edge, resulting in a reduced hole concentration at GBs^8^,^9^,^10^. The potential landscape around GBs at the surface of CIGS films has been measured extensively by Kelvin probe force microscopy (KPFM), finding band bending of different sizes and directions around GBs, mostly attributed to charged defect states^11^,^12^,^13^,^14^,^15^. Other scanning probe microscopy studies have explained electronic GB properties by a valence band offset^16^,^17^,^18^. The role of Na at GBs has received particular attention, and its presence has been directly observed at GBs^19^. It has long been known that the alkali element Na is essential for obtaining high efficiencies^20^,^21^, where Na can be introduced by various strategies^22^. Interestingly, the recent efficiency increases in CIGS solar cells have been achieved by a potassium fluoride post-deposition treatment (KF-PDT) applied after the deposition of the CIGS absorber^23^,^24^. The reasons for the beneficial effect of the KF-PDT are currently being investigated. An increase in the net carrier concentration as a result of the presence of K has been reported^25^,^26^, leading to an increase in the open circuit voltage. This has been attributed to the reduction of compensating donors, similar to what has previously been described for Na^27^. A strong Cu and Ga depletion resulting from the KF-PDT has been observed at the surface of CIGS by x-ray photoelectron spectroscopy (XPS)^23^,^28^,^29^,^30^. An ion exchange mechanism has been proposed, where K replaces Na in the near surface region of the CIGS^29^. These observations led to the proposal of a modified surface, consisting in In_2_Se_3_ or KInSe_2_^30^. A widened band gap at the surface has been reported, where a lowering of the valence band edge has been assigned to result from the Cu depletion^28^,^30^, in agreement with theoretical predictions^8^,^31^.

While most studies to date have focused on the surface and close to surface effect of the KF-PDT, it had been speculated that K might also be found at grain boundaries (GBs)^28^,^29^. Very recently, atom probe tomography measurements have in fact observed an increased K concentration at grain boundaries^32^,^33^. Here we report the first study of the effect of the KF-PDT on the electronic GB properties. We present a comparative KPFM study of CIGS with and without the KF-PDT. Furthermore, we also studied the initial formation of CdS on the CIGS surface and its effect on the surface electronic properties.

## Experimental

We studied a set of four different samples, consisting in CIGS without and with a KF-PDT and without and with a thin CdS layer, denominated: CIGS, KF-CIGS, CIGS/CdS and KF-CIGS/CdS, respectively. CIGS absorber layers were co-evaporated on Mo-coated soda-lime glass (SLG) following the 3-stage process^34^. A standard KF-PDT, consisting in evaporating 150 Å of KF at 0.1 Å/s under Se atmosphere directly onto the CIGS absorber heated at 350 °C, was applied for KF-CIGS and KF-CIGS/CdS samples. The CdS buffer layer was grown by chemical bath deposition in an open reactor heated at 60 °C, kept opened and containing ammonia (20 ml, 1 mol/l), cadmium acetate dihydrate (12 ml, 2.6 × 10^−3^ mol/l) and thiourea (12 ml, 9.5 10^−2^ mol/l). CIGS/CdS and KF-CIGS/CdS were dipped for one minute in the bath and then rinsed with deionized water and dried with N_2_ gas. CIGS and KF-CIGS were also used to complete solar cell devices to verify the beneficial effect of the treatment. 60 nm-thick CdS (7 minutes in the same bath), as well as ZnO/ZnO:Al bilayers and contact grids were deposited onto these absorbers following the baseline of the IMN^35^. Table 1 shows the photovoltaic parameters of these devices. The KF-treated device exhibits a higher open circuit voltage (V_oc_) than the KF-free one, while fill factor (FF) and short circuit current density (J_sc_) have similar values, yielding an important increase of the conversion efficiency ( + 8 rel%).

All samples for the KPFM study were kept under N_2_ atmosphere after the growth to reduce any uncontrolled modification of the surfaces. They were taken from the N_2_ atmosphere immediately prior to the KPFM experiments. KPFM measurements were performed in a Bruker Dimension Icon atomic force microscope (AFM) operated in ambient environment. We used Pt/Ir-coated cantilevers (PPP-EFM Nanosensors^TM^) with a nominal tip radius of 25 nm, spring constant of 2.8 N/m, and 75 kHz resonance frequency. To ensure comparability of the results the tips were calibrated using an Au-coated Si sample. The surface potential was measured using amplitude-modulated KPFM in a dual pass technique. In the first pass the topography line profile is mapped by tapping mode AFM. The topography is traced at a lift height of 5 nm in the second pass, during which an AC-bias voltage (V_AC_ = 500 mV) is applied to the tip at the mechanical resonance *f*_*0*_ of the cantilever. The oscillating electrostatic forces are minimized by applying a compensating V_DC_ to the tip. The contact potential difference (CPD) is then determined as V_CPD_ = −V_DC_ = e(Φ_sample_ − Φ_tip_), where Φ is the work function. For the analysis of the CPD variation across GBs, we identified the GB based on the topography image^15^ and analyzed a CPD line profile perpendicular to the GB averaging over an about 150 nm wide segment of the GB.

## Results

**Figure 1:**
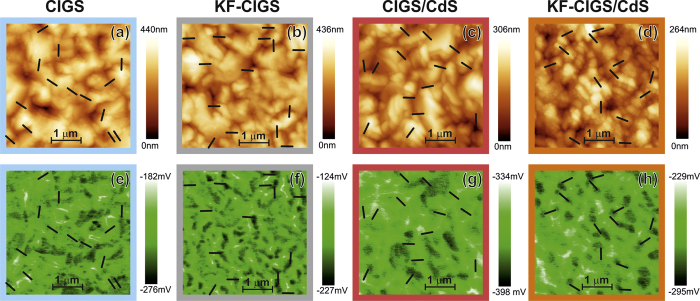
KPFM measurements on the four samples showing (**a**–**d**) topography and (**e**–**h**) simultaneously recorded CPD images of CIGS, KF-CIGS, CIGS/CdS and KF-CIGS/CdS.

The topography of all samples appears similar (Fig. 1). Some structures of order of a few tens of nanometers can be seen in all samples, decorating mostly the surface/facets of the grains (see Supplementary Information). These small structures/clusters appear to be more abundant in the CIGS samples in comparison with the CdS-coated ones. The CPD images also provide a fairly similar contrast between the samples.

**Figure 2:**
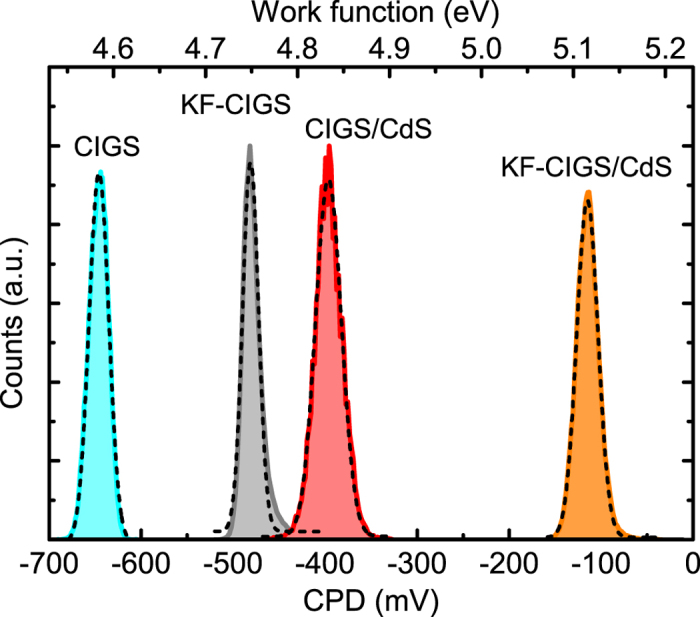
CPD distribution obtained for the four investigated samples. Dashed lines represent Gaussian fits of the histograms. The upper axis gives the work function obtained using the calibration of the AFM tip on the Au/Si reference sample.

To compare the electronic surface properties of the samples, the distributions of CPD values are shown as histograms in Fig. 2. The CPD histograms correspond to the initial KPFM measurement of each sample, taken immediately after removal from the N_2_ atmosphere. To ensure the stability of the surface electronic properties, we monitored the development of the CPD with time over a period of ~10 hours by repeated KPFM experiments with intermediate calibrations of the AFM tip on an Au/Si reference sample (Fig. S2, Supplementary Information).

For quantitative evaluation, each histogram in Fig. 2 was fitted by a Gaussian distribution, from which an average CPD and a distribution of CPD values (full-width at half maximum (FWHM)) are determined. The values are summarized in Table 2. It is seen that the KF-PDT increases the surface work function by ~160 meV in case of the CIGS layers and by ~280 meV in case of CIGS/CdS samples. The FWHM values, representative of the distribution of the CPD across the sample, show similar values for all samples and can be attributed to the CPD change at GBs (see below) and other surface inhomogeneities. The deposition of the thin CdS also results in an increase of the work function, ~250 meV in the case of CIGS and ~370 meV for the KF-CIGS.

**Figure 3:**
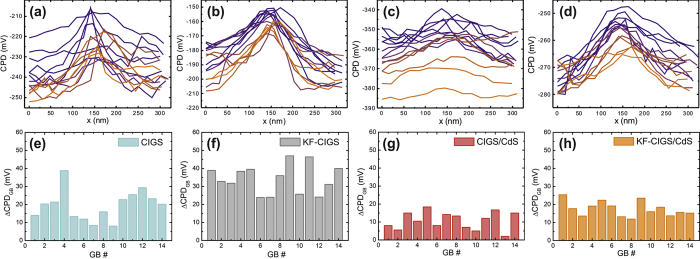
CPD variation across GBs for the four studied samples. (**a**–**d**) Line profiles of all analyzed GBs per sample and (**e**–**h**) CPD variation (ΔCPD_GB_), across the GBs extracted from the line profiles (see text for details).

**Figure 4:**
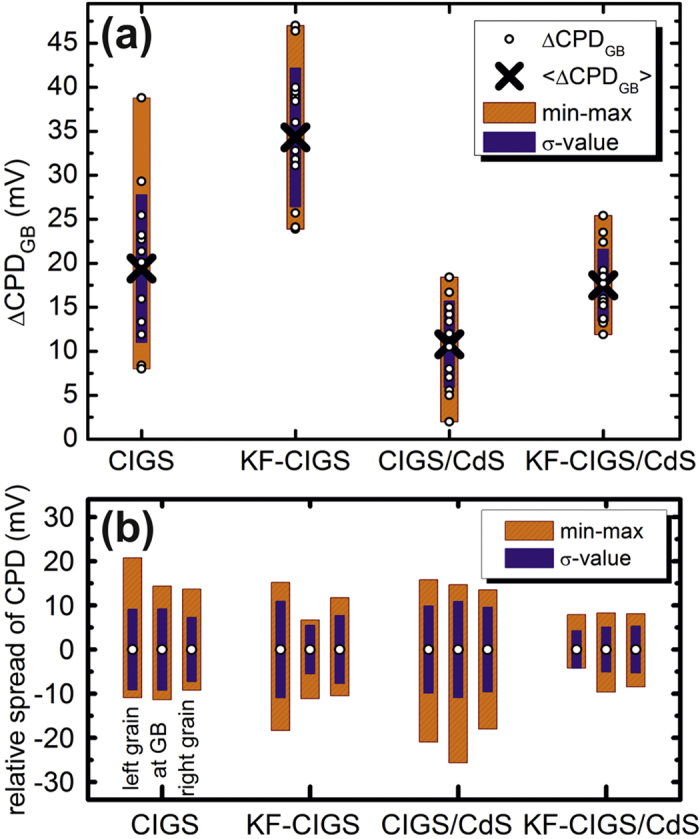
(**a**) The CPD variation across the GBs (ΔCPD_GB_) in all samples. The individual values, ΔCPD_GB_, of all GBs are indicated by the small open circles. The average value, <ΔCPD_GB_> , is indicated by the cross, the minimum-to-maximum spread by the wider orange box, and the standard deviation by the narrower purple box. (**b**) Spread and standard deviation of the CPD values at the GBs and on the grain surface to the left and right of the GBs, shown relative to the average CPD to facilitate comparison.

To analyze in detail the CPD variation at GBs, we considered 14 GBs for each sample (Fig. 3). The location of the GBs (indicated in Fig. 1) was identified based on the topography images^15^ to ensure their selection independent of specific CPD changes. Nearly all analyzed GBs show an increase of the CPD at the GB (Fig. 3a–d). The variation ΔCPD_GB_ was determined by analyzing the difference between the CPD value at the GB and the average CPD value of the grain surfaces on both sides of the GBs; these values are illustrated in Fig. 3e–h. Two observations can be made from these plots, the short CdS deposition attenuates the CPD variation at GBs and the samples with KF-PDT show a smaller spread of the CPD variations at the GBs. These observations are analyzed statistically in Fig. 4a, showing a summary of the analysis of the CPD variation at the GBs of all investigated samples. Several observations can be made. (i) The average ΔCPD_GB_ (cross symbol) is larger for samples with a KF-PDT, compared with the untreated CIGS samples. This applies for samples with and without the CdS deposition. (ii) Nevertheless, the spread of the ΔCPD_GB_ values (orange and purple bars) is smaller for samples with KF-PDT, again, this applies for samples with and without the CdS layer. (iii) The CdS deposition reduces the value of ΔCPD_GB_ for both, CIGS with and without KF-PDT. (iv) The CdS deposition also reduces the spread of the ΔCPD_GB_ values, again, for samples with and without the KF-PDT. These observations lead us to the following conclusions: (a) the KF-PDT increases the CPD at the GBs and makes it more homogeneous, and (b) the CdS deposition “screens” the CPD variation at the GBs.

From the line profiles of all GBs shown in Fig. 3a–d, it can be observed that the variation of the CPD value at the GBs is smaller for the KF-CIGS samples compared with the CIGS samples without the treatment. This observation is quantified in Fig. 4b, showing the spread of the CPD values at the GB and those on the grain surfaces to the left and right side of the GB. It is clearly observed that the spread of the CPD values at the GBs is smaller for the KF-CIGS samples. The observations from the results in Fig. 4 indicate that the KF-PDT leads to more homogeneous GB electronic properties.

## Discussion

The observed overall increase in the work function due to the KF-PDT (about +160 meV for CIGS and +280 meV for CIGS/CdS) can result from (i) a surface dipole (by means of a change in electron affinity), (ii) a different surface material (by means of a direct change in the work function), (iii) a different band bending (by means of different surface defects or surface charging), (iv) a different charge carrier concentration (leading to a change in the Fermi level), or (v) a band gap widening (in the case of shift of the conduction band minimum). The increase in CPD is in agreement with the observations of Handick *et al*.^30^, who report indications of an In_2_Se_3_ or a KInSe_2_ surface composition and a significantly increased band gap. In any case, a change in the work function of the CIGS layer should affect the band alignment to the CdS layer. The short deposition of CdS for 1 min does not lead to the growth of a CdS film sufficient to form a pn junction. For a sufficiently thick CdS deposition, a lowering of the work function could be expected in accordance with the n-type character of CdS. The increase of the work function upon the 1-min deposition of CdS therefore indicates a surface modification; this might be a surface cleaning or the formation of a different surface compound, e.g. CdIn_2_S_4_, as proposed recently by Lepetit *et al*.^36^.

**Figure 5:**
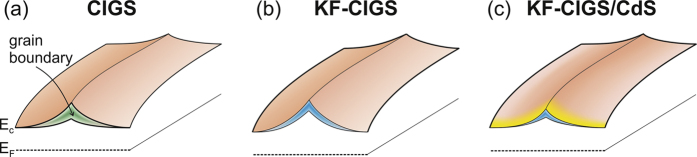
Schematic band diagrams illustrating the electronic structure around grain boundaries for (**a**) CIGS, (**b**) KF-CIGS, and (**c**) KF-CIGS/CdS. The larger CPD at GBs for KF-CIGS is illustrated, as well as the smaller variation (blue) of the ΔCPD_GB_ for the KF-CIGS and the KF-CIGS/CdS with respect to that of the CIGS sample (green).

The main result of our work relates to the electronic properties of the GBs. We observed that the KF-PDT increases the upward band bending at the GBs (Fig. 5). This increase is compatible with the observed general increase of the work function and indicates that the effect of K at the GBs is enhanced. In fact, recent atom probe tomography experiments have directly observed an increase in the K concentration at GBs^32^,^33^. Two-dimensional device simulations have shown that sufficient band bending at GBs can lead to improved device characteristics^37^,^38^. We note that the measured ΔCPD_GB_ values need to be corrected considering the well-known averaging effect of KPFM^39^,^40^. According to^15^,^41^, a correction factor of about 2–5 should be applied considering the lift height of 5 nm. Nevertheless, it should be noted that the increase of band bending at the GBs is smaller than the overall increase of the CPD on the surface. We would also like to point out that while KPFM is a surface characterization technique, the potential variations measured do reflect the potential in the near-surface region. KPFM measures and compensates the local electrostatic field. In the case of charges localized below the surface, their electric field will be screened by the surrounding material and the screening length will depend on the charge-carrier concentration. Therefore, the observed potential variations are expected to be representative for the near-surface region, which is the location of the charge-separating junction.

In addition to the increased upward band bending at the GBs, we consider it at least equally important that the KF-PDT results in a more homogeneous CPD distribution at the GBs (Fig. 5). A narrow distribution of CPD values should improve the junction properties at the GBs and could correspond to reduced voltage losses due to different band alignments at different GBs, in agreement with the improved V_oc_ of the KF-treated CIGS device (Table 1). Moreover, the initial CdS layer formation results in a further reduced variation at the GBs, making the band alignment even more homogeneous. This observation is in agreement with the favorable Cd diffusion into the CIGS layer and GBs, controlling the band bending by the formation of Cd point defects, as has been proposed by Pianezzi *et al*.^26^. Theoretical studies support such n-type doping of Cd in CIGS^42^,^43^.

## Conclusion

So far, the positive effect of the KF-PDT on CIGS solar cells was attributed to modifications of the doping concentration and the interface forming the pn-junction. Our results indicate that the KF-PDT additionally contributes to more homogeneous electronic properties of the GBs at the CIGS surface, which should improve the band alignment at the GBs, reducing losses in the open circuit voltage and leading to an efficiency enhancement. In view of the general relevance of the electronic activity in GBs and its impact on the efficiency of polycrystalline solar cell materials (e.g. CdTe and perovskites), an improved understanding of the beneficial modification of GB electronic properties might lead to the development of strategies to improve GB properties also in other solar cell and electronic materials.

## Additional Information

**How to cite this article**: Nicoara, N. *et al*. Effect of the KF post-deposition treatment on grain boundary properties in Cu(In, Ga)Se_2_ thin films. *Sci. Rep.* **7**, 41361; doi: 10.1038/srep41361 (2017).

**Publisher's note:** Springer Nature remains neutral with regard to jurisdictional claims in published maps and institutional affiliations.

## Supplementary Material

Supplementary Information

## Figures and Tables

**Table 1:** Device parameters of complete solar cells without and with a KF-PDT.

Sample	V_oc_ (mV)	Fill Factor (%)	J_sc_ (mA cm^−2^)	Efficiency (%)
KF-free device	655 ± 1	75.3 ± 0.1	33.7 ± 0.1	16.6 ± 0.3
KF-treated device	720 ± 1	74.7 ± 0.1	33.5 ± 0.1	18.0 ± 0.3

**Table 2:** CPD average and FWHM values obtained from the Gaussian fits to the CPD histograms of the four studied samples.

	CIGS	KF-CIGS	CIGS/CdS	KF-CIGS/CdS
CPD_mean_ (mV)	−645	−481	−396	−115
FWHM (mV)	25	20	33	27
Φ (eV)	4.59	4.75	4.82	5.11
<ΔCPD_GB_> (mV)	19.4 ± 8.4	34.3 ± 7.9	10.8 ± 4.9	17.5 ± 4.1

Using the calibration on an Au/Si sample, the work function was estimated, assuming Φ_Au_ = 5.1 eV. The average CPD changes at the grain boundaries, <ΔCPD_GB_>, are also given (see Fig. 4a).

## References

[b1] National Renewable Energy Laboratory. Best Research Cell Efficiencies, http://www.nrel.gov/pv/assets/images/efficiency_chart.jpg. (Date of access: 31/10/2016).

[b2] Jackson P. . Effects of heavy alkali elements in Cu(In,Ga)Se2 solar cells with efficiencies up to 22.6%. Phys. Status Solidi RRL. doi: 10.1002/pssr.201600199 (2016).

[b3] Poplawsky J. D. . Direct Imaging of Cl- and Cu-Induced Short-Circuit Efficiency Changes in CdTe Solar Cells. Adv. Energy Mater. 4, 1400454 (2014).

[b4] Zhang L. . Effect of Copassivation of Cl and Cu on CdTe Grain Boundaries. Phys. Rev. Lett. 101, 155501 (2008). 18999610 10.1103/PhysRevLett.101.155501

[b5] Shao Y., Xiao Z., Bi C., Yuan Y. & Huang J. Origin and elimination of photocurrent hysteresis by fullerene passivation in CH_3_NH_3_PbI_3_ planar heterojunction solar cells. Nat. Commun. 5, 5784 (2014). 25503258 10.1038/ncomms6784

[b6] Zhang W., Eperon G. E. & Snaith H. J. Metal halide perovskites for energy applications. Nat. Energy 1, 16048 (2016).

[b7] deQuilettes D. W. . Impact of microstructure on local carrier lifetime in perovskite solar cells. Science 348, 683–686 (2015). 25931446 10.1126/science.aaa5333

[b8] Persson C. & Zunger A. Anomalous grain boundary physics in polycrystalline CuInSe_2_: the existence of a hole barrier. Phys. Rev. Lett. 91, 266401–266404 (2003). 14754073 10.1103/PhysRevLett.91.266401

[b9] Siebentritt S. . Evidence for a neutral grain-boundary barrier in chalcopyrites. Phys. Rev. Lett. 97, 146601–146604 (2006). 17155277 10.1103/PhysRevLett.97.146601

[b10] Hafemeister M., Siebentritt S., Albert J., Lux-Steiner M. C. & Sadewasser S. Large neutral barrier at grain boundaries in chalcopyrite thin films. Phys. Rev. Lett. 104, 196602–196606 (2010). 20866985 10.1103/PhysRevLett.104.196602

[b11] Sadewasser S. . Kelvin probe force microscopy for the nanoscale characterization of chalcopyrite solar cell materials and devices. Thin Solid Films. 431–432, 257–261 (2003).

[b12] Jiang C.-S. . Does the local built-in potential on grain boundaries of Cu(In,Ga)Se_2_ thin films benefit photovoltaic performance of the device? Appl. Phys. Lett. 85, 2625–2627 (2004).

[b13] Fuertes Marrón D., Sadewasser S., Meeder A., Glatzel T. & Lux-Steiner M. C. Electrical activity at grain boundaries of Cu(In,Ga)Se_2_ thin films. Phys. Rev. B 71, 033306–033310 (2005).

[b14] Baier R. . Electronic properties of grain boundaries in Cu(In,Ga)Se_2_ thin films for various Ga-contents. Sol. Energy Mater. Sol. Cells. 103, 86–92 (2012).

[b15] Baier R., Leendertz C., Abou-Ras D., Lux-Steiner M. C. & Sadewasser S. Properties of electronic potential barriers at grain boundaries in Cu(In,Ga)Se_2_ thin films, Sol. Energy Mat. Sol. Cells. 130, 124–131 (2014).

[b16] Azulay D. . Current routes in polycrystalline CuInSe_2_ and Cu(In,Ga)Se_2_ films. Sol. Energy Mat. Sol. Cells. 91, 85–90 (2007).

[b17] Yan Y. . Electrically Benign Behavior of Grain Boundaries in Polycrystalline CuInSe_2_ Films. Phys. Rev. Lett. 99, 235504–235508 (2007). 18233382 10.1103/PhysRevLett.99.235504

[b18] Sadewasser S. . Nanometer-scale electronic and microstructural properties of grain boundaries in Cu(In,Ga)Se_2_, Thin Solid Films. 519, 7341–7346 (2011).

[b19] Cadel E., Barreau N., Kessler J. & Pareige P. Atom probe study of sodium distribution in polycrystalline Cu(In,Ga)Se_2_ thin film. Acta Mater. 58, 2634–2637 (2010).

[b20] Hedström J., Ohlsen H., Bodegård M., Kylner A. & Stolt L. ZnO/CdS/Cu(In,Ga)Se_2_ thin film solar cells with improved performance. *Photovoltaic Specialists Conference, Conference Record of the Twenty Third IEEE*, 364–371 (1993).

[b21] Siebentritt S., Igalson M., Persson C. & Lany S. The electronic structure of chalcopyrites - bands, point defects and grain boundaries. Prog. Photovolt. Res. Appl. 18, 390–410 (2010).

[b22] Salomé P., Rodriguez-Alvarez H. & Sadewasser S. Incorporation of alkali metals in chalcogenide solar cells. Sol. Energy Mat. Sol. Cells. 143, 9–20 (2015).

[b23] Chirila A. . Potassium-induced surface modification of Cu(In,Ga)Se_2_ thin films for high-efficiency solar cells. Nature Mat. 12, 1107–1111 (2013). 10.1038/nmat3789 24185758

[b24] Jackson P. . Properties of Cu(In,Ga)Se_2_ solar cells with new record efficiencies up to 21.7%. Phys. Status Solidi RRL. 9, 28−31 (2015).

[b25] Laemmle A., Wuerz R. & Powalla M. Efficiency enhancement of Cu(In,Ga)Se_2_ thin-film solar cells by a post-deposition treatment with potassium fluoride. Phys. Status Solidi RRL. 7, 631–634 (2013).

[b26] Pianezzi F. . Unveiling the Effects of Post-deposition Treatment with Different Alkaline Elements on the Electronic Properties of CIGS Thin Film Solar Cells. Phys. Chem. Chem. Phys. 16, 8843−8851 (2014). 24675872 10.1039/c4cp00614c

[b27] Ruckh M. . Influence of substrates on the electrical properties of Cu(In,Ga)Se_2_ thin films. Solar Energy Materials and Solar Cells 41/42, 335–343 (1996).

[b28] P. Pistro . Experimental indication for band gap widening of chalcopyrite solar cell absorbers after potassium fluoride treatment. Appl. Phys. Lett. 105, 063901–063904 (2014).

[b29] Reinhard P. . Features of KF and NaF Postdeposition Treatments of Cu(In,Ga)Se_2_ Absorbers for High Efficiency Thin Film Solar Cells. Chem. Mater. 27, 5755−5764 (2015).

[b30] Handick E. . Potassium Postdeposition Treatment-Induced Band Gap Widening at Cu(In,Ga)Se_2_ Surfaces − Reason for Performance Leap? ACS Appl. Mater. Interfaces 7, 27414−27420 (2015). 26633568 10.1021/acsami.5b09231

[b31] Jaffe J. E. & Zunger A. Defect-induced nonpolar-to-polar transition at the surface of chalcopyrite semiconductors. Phys. Rev. B 64, 241304(R)- 241308 (2001).

[b32] Stokes A., Al-Jassim M. & Gorman B. Semi-statistical Atom Probe Tomography Analysis of Thin Film Grain Boundaries. Microscopy and Microanalysis 22, 644–645 (2016).

[b33] Stokes A., Al-Jassim M., Diercks D. & Gorman B. Alkali segregation and matrix concentrations in thin film Cu(In,Ga)Se_2_ at targeted interfaces characterized in 3-D at the nanoscale. *Proc. IEEE 42*^*nd*^ *Photovoltaic Specialist Conference (PVSC)*, New Orleans, LA, 1-4 (2015).

[b34] Ramanathan K. . Properties of 19.2% efficiency ZnO/CdS/CuInGaSe_2_ thin-film solar cells. Prog. Photovolt. Res. Appl. 11, 225–230 (2003).

[b35] Bommersbach P. . Influence of Mo back contact porosity on co-evaporated Cu(In,Ga)Se_2_ thin film properties and related solar cell. Prog. Photovolt. Res. Appl. 21, 332–343 (2013).

[b36] Lepetit T. Influence of KF post deposition treatment on the polycrystalline Cu(In,Ga)Se_2_/CdS heterojunction formation for photovoltaic application. Ph.D. dissertation, Univ. Nantes, Nantes, France, doi: 10.13140/RG.2.1.3451.6242 (2015).

[b37] Gloeckler M., Sites J. R. & Metzger W. K. Grain-boundary recombination in Cu(In,Ga)Se_2_ solar cells. J. Appl. Phys. 98, 113704–113714 (2005).

[b38] Taretto K., Rau U. & Werner J. H. Numerical simulation of grain boundary effects in Cu(In,Ga)Se_2_ thin-film solar cells. Thin Solid Films 480–481, 8–12 (2005).

[b39] Zerweck U., Loppacher C., Otto T., Grafström S. & Eng L. M. Accuracy and resolution limits of Kelvin probe force microscopy. Phys. Rev. B 71, 125424–125433 (2005).

[b40] Leendertz C., Streicher F., Lux-Steiner M. C. & Sadewasser S. Evaluation of Kelvin probe force microscopy for imaging grain boundaries in chalcopyrite thin films. Appl. Phys. Lett. 89, 113120–113123 (2006).

[b41] Baier R., Leendertz C., Lux-Steiner M. C. & Sadewasser S. Toward quantitative Kelvin probe force microscopy of nanoscale potential distributions. Phys. Rev. B. 85, 165436–165442 (2012).

[b42] Persson C., Zhao Y.-J., Lany S. & Zunger A. N-type doping of CuInSe_2_ and CuGaSe_2_. Phys. Rev. B 72, 035211–035225 (2005).

[b43] Kiss J., Gruhn Th., Roma G. & Felser C. Theoretical Study on the Diffusion Mechanism of Cd in the Cu-Poor Phase of CuInSe_2_ Solar Cell Material. J. Phys. Chem. C 117, 25933−25938 (2013).

