# Supplementary material for: Effect of the KF post-deposition treatment on grain boundary properties in Cu(In, Ga)Se2 thin films
Source: Sci Rep. 2017 Jan 27;7:41361. doi: 10.1038/srep41361 (PMC5269666; doi:10.1038/srep41361)
Supplement: Supplementary Information [file srep41361-s1.pdf]

Effect of the KF post-deposition treatment on grain boundary properties in $Cu(In, Ga)Se_2$ thin films

N. Nicoara[1], Th. Lepetit[2], L. Arzel[2], S. Harel[2], N. Barreau[2], and S. Sadewasser[1]

[1] INL – International Iberian Nanotechnology Laboratory, Av. Mestre José Veiga s/n, 4715-330 Braga, Portugal

[2] Institut des Matériaux Jean Rouxel (IMN) - UMR6502, Université de Nantes, CNRS, 2 rue de la Houssinière, BP 32229, 44322 Nantes Cedex 3, France

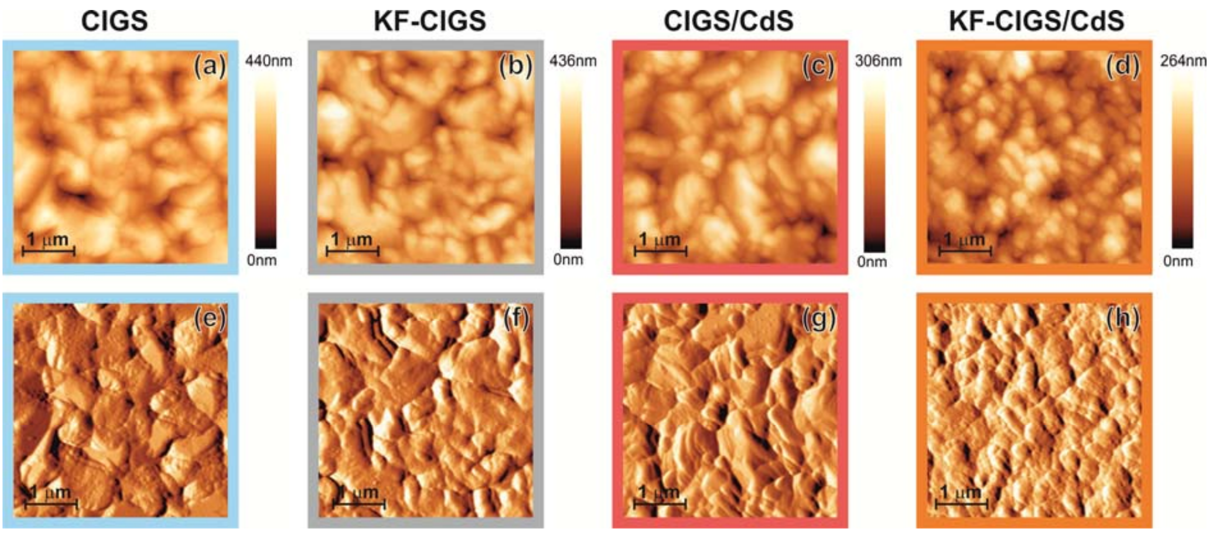

Fig. S1: *KPFM measurements of CIGS, KF-CIGS, CIGS/CdS, and KF-CIGS/CdS showing (a-d) topography and (e-h) derivative of the topography to enhance small height variations.*

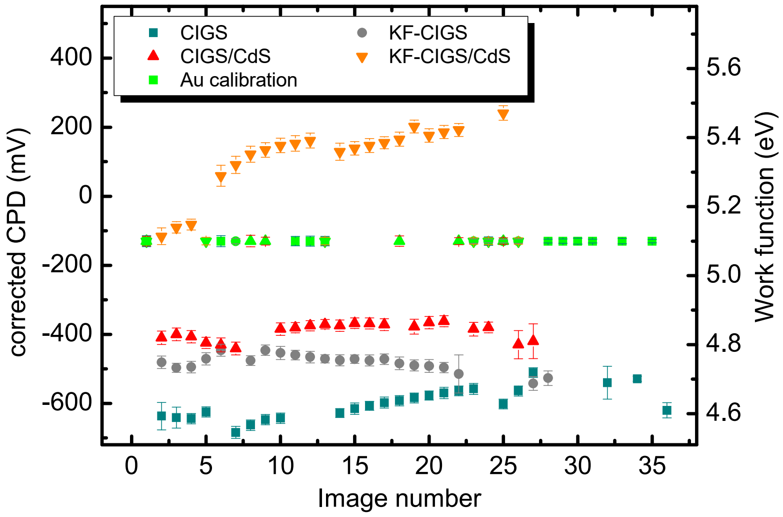

Fig. S2: *CPD values determined for CIGS, KF-CIGS, CIGS/CdS, and KF-CIGS/CdS as a function of time in air. Each image takes about 15 minutes acquisition time. Samples were removed from a protective N_2 atmosphere immediately prior to the first KPFM measurement. To ensure comparability, the AFM tip was calibrated repeatedly on an Au/Si reference sample, which was assumed to have a constant work function of Φ_{Au} = 5.1 eV. Variations in the CPD measurements are assigned to lateral variations of the CPD.*
